# Education and Attitudes Toward Migration in a Cross Country Perspective

**DOI:** 10.3389/fpsyg.2019.02224

**Published:** 2019-10-18

**Authors:** Francesca Borgonovi, Artur Pokropek

**Affiliations:** ^1^Department of Social Science, Institute of Education, University College London, London, United Kingdom; ^2^Institute of Philosophy and Sociology, Polish Academy of Sciences, Warsaw, Poland

**Keywords:** opposition to migration, European Social Survey, education, invariance testing, measurement invariance, threat, cross-country

## Abstract

The paper examines the dynamics of native populations’ opposition to migration and the role of education in shaping such opposition in European countries using data from the last four editions of the European Social Survey between years 2010 and 2016. We examine both the direct association between education and opposition to migration as well as the mediated association that occurs through feelings of threat. We test for measurement equivalence across countries and years of the two latent constructs in our analyses (opposition to migration and feelings of threat) by applying sequential methods used in alignment optimization to identify partial equivalence and check the level of approximate measurement invariance using BSEM modeling. Our results indicate that the opposition to migration and the feelings of threat scales achieve metric invariance but not scalar invariance in cross-country comparisons. At the substantive level, our findings suggest that better educated individuals express lower opposition to migration than the poorly educated and that as much as 60% of education differentials in opposition to migration are due to the mediated effect through feelings of threat. The high degree of heterogeneity in associations both across countries and over time are, in part, explained by the presence of foreign-born populations and living standards in a country and time point.

## Introduction

An estimated 4.9 million migrants arrived in European countries in 2015 (Eurostat, 2018) and while this figure was part of a long and steady upward trend in the share of foreign-born populations residing in European countries, 2015 figures represented a sudden and sizable increase over the 4 million of arrivals registered in 2014 (Eurostat, 2018). Migration flows, particularly sudden increases in the number of new arrivals, can create difficulties for host communities. However, they also represent an opportunity for countries that face aging native-born populations and the associated threat of labor and skills shortages (OECD, 2018). The ability of societies to withstand the pressures on social cohesion posed by migration flows depends on the long-term integration of immigrants, which reflects the host community’s capacity to facilitate the settlement of new arrivals as well as immigrants’ own capacity to adapt and become part of both labor markets and social networks in countries of destinations (OECD, 2018).

Education is often considered an important element for promoting long-term integration processes because it enables immigrants to acquire skills that will lead them to enter the labor market, and because education systems can help migrants understand the culture and the traditions of their country of destination. However, education can also play an important role in shaping the attitudes native populations hold toward immigrants. Migration in fact requires both migrants and natives to undergo a process of acculturation, particularly when the size of the migrant group is large (Berry, 1997). Acculturation has been defined as “culture change that results from continuous, first-hand contact between two distinct cultural groups” (Redfield et al., 1936).

The literature has identified three key mechanisms that drive the formation of native populations’ attitudes toward migration: competition over social and economic resources (Blumer, 1958; Bobo, 1988; Olzak, 1992), threat to the cultural and national homogeneity of society (Fetzer, 2000; Castles and Miller, 2003), and prejudice (Pettigrew and Meertens, 1995; Pettigrew, 1998; Vala et al., 2008). Education can importantly shape individuals’ perceptions of economic threat, cultural threat and prejudice (Schneider, 2008) and, through such effects, might shape individuals’ attitudes toward migration.

The association between education and attitudes toward migration has been studied extensively. Studies have indicated that such association varies across countries and contexts (Borgonovi, 2012; d’Hombres and Nunziata, 2016). Nonetheless, it remains unknown to what extent education promotes positive attitudes toward migration because of direct socialization mechanisms or because of an indirect effect due to a reduction in feelings of threat and prejudice. Furthermore, given rapid changes both in the number of foreign-born populations in European countries, as well as their composition in recent years, it is important to examine not only to what extent the mechanisms shaping the association between education and opposition to migration differ across countries but also how they evolve over time, and if such differences are systematically related to economic factors such as level of income inequality or economic development or to the stocks of migration communities. Finally, many existing studies examining education differentials in either threat perceptions or opposition to migration attitudes do not investigate the comparability across countries and over time of key indicators.

In this paper we use data on countries that participated in the last four rounds of the European Social Survey (rounds 5, 6, 7, and 8), a large and nationally representative survey capturing attitudes toward migration of individuals aged 15 and above residing in Europe. Round 5 was implemented in 2010, round 6 was implemented in 2012, round 7 was implemented in 2014, just before the migration crisis hit European countries, and round 8 was implemented in 2016. Our analysis focuses on the eighteen countries that participated in rounds 5, 6, 7, and 8 of the European Social Survey: Belgium, Switzerland, Czechia, Germany, Estonia, Spain, Finland, France, Great Britain, Hungary, Ireland, Israel, Lithuania, Netherlands, Norway, Poland, Portugal, Slovenia.

The aim of the paper is threefold. First, we establish if measures of attitudes toward migration and individuals’ perceptions of threat that are common to the four rounds of the European Social Survey can be compared across countries and over time. Second, we identify the extent to which the relationship between education and attitudes toward migration is mediated by feelings of threat. Third, we consider if variations across countries and over time in the association between education and attitudes toward migration and the extent to which this association is mediated by feelings of threat depends on contextual features, most notably level of economic development (as measured by per capita Gross Domestic Product - GDP), level of income inequality (as measured by the Gini index), migration flows (as measured by the change in the number of foreign-born individuals who reside in a country between the study year t and t-2) and the percentage of people born in a foreign country (as an indication for the overall level of migration).

## Theory and Hypotheses

At the individual level, empirical research has documented a strong relationship between educational attainment and attitudes toward migration: better educated individuals tend to display more openness toward migrants than those with fewer educational qualifications (see for example, Quillian, 1995; Scheepers et al., 2002; Kunovich, 2004; Semyonov et al., 2006; Gesthuizen et al., 2008). However, few studies have examined cross-country variations in the relationship between education and attitudes toward migration. Quillian (1995), Scheepers et al. (2002), Kunovich (2004), Borgonovi (2012), and d’Hombres and Nunziata (2016) represent important exceptions.

Even less is known about why and how education matters, in other words what are the underlying social, psychological and cognitive processes that determine an association between education and attitudes toward migration and if the strength of the association between education and attitudes toward migration depends on the conditions and circumstances individuals experience.

Group threat theory provides a useful framework to identify factors that shape the development of attitudes toward migration, how such attitudes may differ depending on individuals’ educational attainment, and external conditions such as the size of migrant communities and the economic situation of a country. Group threat theory predicts that members of a group will exhibit feelings of solidarity toward individuals that they consider to be part of their group and negative attitudes toward those who do not. Negative attitudes arise from a perceived threat from out-of-group members to the interest of the group (Blumer, 1958). Group identification and perceived threat induced by out-of-group members are conceptually distinct but can be mutually reinforcing: strong feelings of identification with a group depend, to a great extent, to exposure to out-of-group individuals: “we are what we are because they are not what we are” (Tajfel, 1979; Tajfel and Turner, 1979). Group threat theory essentially maintains that because of the actual or anticipated negative consequences in-group members suffer (or believe they will suffer) because of out-group members, in-group members develop explicit preferences for “denying out-of-group members equality of treatment that out-of-group members may wish to have” (Allport, 1954).

Group threat theory predicts that, other things being equal, the more threatened natives feel by migrants, the more negative their attitudes toward migrants will be (Blumer, 1958; Case et al., 1989; Bobo and Hutchings, 1996; Scheepers et al., 2002; Semyonov et al., 2002, 2004, 2006; Sniderman et al., 2004).

Recent empirical findings show how the attitude toward foreigners in host countries depends on both economic and non-economic factors. Some authors highlight that natives feel threatened by the competition in the labor market that arises from immigration (Scheve and Slaughter, 2001; Mayda, 2006), while other authors stress the importance of non-economic factors, such as racial intolerance and prejudice (Dustmann and Preston, 2001), and how both kinds of factors play a significant role (Citrin et al., 1997; O’Rourke and Sinnott, 2006; Hainmueller and Hiscox, 2010). Dustmann and Preston (2007) suggest that welfare concerns play a more important role than labor market concerns, and that racial and cultural prejudices relate primarily to immigrants from different ethnic backgrounds. We conceive that individuals’ generalized threat is determined by three factors: economic threat, cultural threat and prejudice.

Attitudes toward migration may be driven by the fear (or lack of fear) of labor-market competition from migrants, what is defined in the literature as economic threat. Although the evidence on the net effect of immigration on the wages of native populations is mixed, with some studies estimating a negative effect of immigration on the wages of competing workers (Borjas, 2003), and other studies failing to find adverse effects (D’Amuri et al., 2010; Ottaviano et al., 2013), poorly educated individuals may perceive migrants as potentially substituting them in the labor market (since migrants are, on average, with lower skills than the average native in Europe) while better educated individuals may perceive migrants to bring complementarities to their work (d’Hombres and Nunziata, 2016). The literature indeed suggests that low-skilled native workers are more likely to express feelings of threat (Schneider, 2008), support limits to migration flows or to hold negative attitudes toward migration (Scheve and Slaughter, 2001; Mayda, 2006; O’Rourke and Sinnott, 2006), but also that such effect can only be observed among low-skilled natives who are in the labor market.

Education also fosters individuals’ information processing abilities and, as a result, better educated individuals may be better placed to interpret and evaluate migration phenomena, enabling them to consider the potential long-term positive economic effects that migration can bring to host countries in terms of taxes and social contributions which tend to match or even surpass, the amount of individual benefits that they receive (OECD, 2013).

Cultural (symbolic) threat characterizes the perceived threat native populations feel when they enter in contact with out-of-group members because out-of-group members hold distinct norms, moral and values from their own (Schnapper, 1994; Fetzer, 2000; Stephan et al., 2000; Castles and Miller, 2003). Differences in values, norms and morals threaten the cultural identity of in-group members because individuals’ sense of self and of belonging to a community depends on the articulation of a set of common attitudes and values to which all members of the community subscribe. Group threat theory predicts that when individuals feel that their culture (defined as the organized set of attitudes, values, goals and practices that inform and govern the beliefs and behaviors of a group of people or a society), is threatened by the potential integration of migrants, they will hold more negative attitudes toward migration.

Cultural threat depends both on the level of perceived distinctiveness between in-group and out-groups in attitudes, morals and values (with greater differences being associated with more negative attitudes), individuals’ adherence to a specific and well-defined set of values, morals and attitudes (with greater adoption being associated with greater perceived threat) and the consideration of such values and morals as universally valid (with greater perceived universality being associated with greater perceived threat).

While highly educated individuals have benefited greatly from globalization and the integration of economies and labor markets, individuals with low levels of education have been increasingly left behind (Autor, 2014). The progressive erosion of social status experienced by poorly educated individuals as a result of globalization has led to high levels of anomie among some but also to an increased adherence to the traditional attitudes, values and mores prevalent in their country, and by an increased feeling that such attitudes, values and mores are morally justified and should be followed by all because they are superior to the attitudes, values and mores prevalent in other societies (Sapolsky, 2017).

A final source of component of general threat is prejudice. Prejudice reflects general negative feelings individuals may hold against people who are out-of-group members. Prejudice constitutes a set of socially learned feelings and is usually associated with racial or ethnic diversity (Allport, 1954; Kinder and Sears, 1981; Sears and Kinder, 1985; Katz, 1991). It is defined as a collection of negative attitudes “toward a socially defined group and toward any person perceived to be a member of that group” (Ashmore, 1970, p. 253) or as “antipathy based on faulty and inflexible generalization” (Allport, 1954, p. 7). Formal education and schooling, given the strong emphasis that they have on equipping individuals with information processing abilities, should reduce the incidence of prejudice. Contrary to economic or cultural threat, prejudice is not rooted into the economic or cultural institutions of a country but, rather in irrational generalizations.

Schneider (2008) indicated that in the early 2000s in Europe education was importantly associated with feelings of threat: individuals who attended school for longer were less likely to express feelings of cultural threat and economic threat than similar individuals who attended school for a smaller number of years. However, Schneider did not examine the extent to which feelings of threat map onto opposition to migration and did not establish if the association between education and feelings of threat differs across countries. We hypothesize that better educated individuals will experience lower feelings of generalized threat and therefore will report more positive attitudes toward migration than those who attended school for less because of a strong association between feelings and attitudes.

Although we expect that most of the association between education and attitudes toward migration will be mediated by perceived economic threat, cultural threat and prejudice, education may also be directly associated with attitudes toward migration. The direct association between education and attitudes toward migration may reflect the intergenerational transmission of education and differences in the socialization processes experienced by individuals with highly educated and poorly educated parents. Children internalize from their parents societal norms, attitudes and values (Putnam, 1993; Uslaner, 2002; Stolle and Hooghe, 2004; Johnson and Dawes, 2016) and discuss political and social issues with their parents and family members (Dostie-Goulet, 2009). There is evidence that parents influence young people’s interest in politics, political participation and political efficacy (Dawson and Prewitt, 1969; Dennis, 1973; Andolina et al., 2003; McIntosh et al., 2007; Dostie-Goulet, 2009). Given past evidence on the positive association between education and the likelihood that individuals will hold favorable attitudes toward migration, better educated parents are more likely to socialize their children into also holding similarly favorable attitudes, an effect that could be magnified by the fact that better educated parents tend to be more engaged with their children and to discuss with them social and political issues while they grow and start to form their own attitudes and opinions (Borgonovi and Montt, 2012).

Because group threat theory predicts that attitudes toward migration depend on perceived threat, it predicts that, other things being equal, the greater the growth in the size of immigrant populations over time is, the greater the perceived threat will be and, as a result, the more negative attitudes toward migrants among native populations will be (Blumer, 1958; Blalock, 1967; Bobo, 1988). However, this prediction holds under equality of conditions. Therefore, observed differences in attitudes toward migration across countries with different levels of migrant populations (or changes in such population over time) may not be in line with group threat theory predictions on a negative association between foreign-born group size and attitudes. A larger group of foreign-born individuals is in fact likely to pose a lower perceived threat in countries and periods characterized by a more favorable economic situation and a society characterized by egalitarian principles (Semyonov et al., 2008).

Furthermore, intergroup contact theory predicts that as the relative size of the foreign-born population increases, members of the two groups will have more opportunities for direct contact and, with contact, perceived threat could be lower. While initially it was considered that intergroup contact would promote positive intergroup attitudes only under optimal conditions (such as the presence of common goals, intergroup co-operation, equal status and authority support) (Allport, 1954) proponents of intergroup contact theory have recently suggested that intergroup contact can promote positive intergroup attitudes even when the optimality of conditions situation is not satisfied (Stein et al., 2000; Pettigrew and Tropp, 2008).

Empirical studies fail to provide conclusive evidence on the association between the size of migrant populations and natives’ attitudes toward migration: some studies indicate that larger foreign-born populations are associated with more negative attitudes (Scheepers et al., 2002; Semyonov et al., 2006), some fail to find an association (Evans and Need, 2002; Coenders et al., 2005; Strabac and Listhaug, 2008) while others find a positive association (Lubbers, 2006). Schneider (2008) found a non-linear association between the size of foreign-born populations and feelings of threat: when the size of foreign-born populations is small, increases in foreign-born populations are associated with increased feelings of threat, but when the size is large, increases in foreign-born populations are associated with a reduction in feelings of threat.

We examine if individuals’ attitudes toward migrants are associated with the change in the percentage of the population who is foreign-born in the recent period (2 years prior to the year in which the respondent was interviewed) as well as the living standards (proxied by GDP per capita in the year in which the respondent was interviewed) and level of income inequality (proxied by the income Gini coefficient in the year in which the respondent was interviewed). Furthermore, we identify if migrant flows, living standards as well as level of income inequality moderate the indirect association of education on attitudes toward migration through general threat.

## Materials and Methods

### The European Social Survey

The European Social Survey (ESS) is an academically driven cross-national survey that has been mapping attitudes and behavioral changes in Europe’s social, political and moral climate since its establishment in 2001. The survey conducts face-to-face interviews every 2 years with newly selected, cross-sectional samples that are representative of all persons above the age of 14 and who are resident within private households in each country. The sample size requested to participating countries is at least 1,500 respondents, although for countries with small populations the number of respondents can be smaller. The first round was conducted in 2002 in 22 countries. Since then around 350,000 face-to-face interviews have been carried out and over 35 countries have participated in at least one ESS round.

The questionnaire consists of a main core section of questions that have been administered in every ESS round and are thus easily comparable over time. These questions were developed following the recommendations made by academic experts who were consulted by the Core Scientific Team during the early planning stages of the ESS. The core modules contain questions aimed at identifying individuals’ attitudes toward the media, health and wellbeing, trust in institutions and governments, education and occupation, social capital and social trust, household circumstances, citizen involvement and democracy, social exclusion, political values and engagement, immigration and crime. In addition to questions on attitudes and dispositions, the ESS contains information on socio-demographic variables such as respondents’ ethnic and immigrant background, household income, level of education, employment and occupational status of the respondent, his/her parents and partner.

In addition to the ‘core’ modules that are administered in each round, multinational teams of researchers based in ESS countries were selected to contribute to the design of additional ‘rotating questionnaires.’ ‘Rotating questionnaires’ that have been administered so far include questions on citizen involvement, health and care, economic morality, family, work and wellbeing, timing of life, personal and social wellbeing, welfare attitudes, ageism, trust in the police and courts, democracy, immigration, social inequalities in health and attitudes to climate change and energy security. Some of these topics have been included in more than one ESS round.

Analyses are based on data from the last four rounds of the European Social Survey (ESS), rounds 5, 6, 7, and 8. Face-to-face interviews were conducted with residents aged 15 or over in 2010 (ESS5), 2012 (ESS6), 2014 (ESS7), and 2016 (ESS8) using multistage probability sampling. The following 18 countries are part of our analysis: Belgium, Switzerland, Czechia, Germany, Estonia, Spain, Finland, France, Great Britain, Hungary, Ireland, Israel, Lithuania, Netherlands, Norway, Poland, Portugal, and Slovenia.

We include in our analysis only individuals who were born in the country in which they resided at the time of the ESS interview and who were aged 25. We run analyses on individuals aged 25 or above because in many countries a significant number of individuals below this age are still in education or may return to study to complete their studies following an interruption and therefore for these individuals the variable measuring schooling may be misspecfied. To ensure that this sample restriction does not lead to bias, we run all analyses using the full sample irrespective of age but excluding individuals who report being still in education. Results are comparable to the ones presented. These restrictions lead to a final working sample of 106,679 individuals. Slightly less than 5% of observations have at least one missing data (mostly in our dependent variables). As this percentage is relatively small and our modeling procedures complex we decided to work only on data with full record of responses applying listwise deletion. This procedure is justify giving small number of missing data (Newman, 2010, 2014) and gave us 101,351 observations as final sample.

### Variable Description

#### Individual Level Variables

We consider our key outcome indicator, individuals’ opposition to migration, to be a latent construct which we measure using three questions in ESS: (1) ‘to what extent do you think [country] should allow people of the same race or ethnic group as most [country] people to come and live here?’ (2) ‘how about people of a different race or ethnic group from most [country]?’ (3) ‘how about people from the poorer countries in Europe?’. Response options were 1 (many), 2 (some), 3 (a few), and 4 (none). Positive values of the index indicate high opposition to migration while low values represent low levels of opposition. We standardized the index to have a distribution with a mean of zero and a standard deviation of one in year 2010 in each country. Standardized values Z = (X-MX2010)/SDX2010 where MX2010 corresponds to the mean of the index for the pooled sample of respondents in year 2010 and SD_X__2010_ corresponds to the standard deviation of the index for the pooled sample of respondents in year 2010.

Our key explanatory factors are individuals’ educational attainment and feelings of generalized threat. Educational attainment was measured through an indicator of the number of years of schooling that the respondent reported having attended.

We also construct a generalized threat index using indicators available in ESS on feelings of economic threat, cultural threat and general prejudice. In the ESS survey each of these indicators is represented by a single item measured on a 10-category scale. Economic threat is measured through responses to the question “would you say it is generally bad or good for [country]’s economy that people come to live here from other countries?”. Cultural threat is measured through responses to the question “would you say that [country]’s cultural life is generally undermined or enriched by people coming to live here from other countries?”. Prejudice is measured through responses to the question “is [country] made a worse or a better place to live by people coming to live here from other countries?”. This index is constructed using the same methodology as opposition to migration (see details in section Materials and Methods). Positive values of the index indicate high level of threat while low values indicate low levels of threat. We standardized the index using the same procedure described for the opposition to migration index.

All models control for age, gender, if the respondent has children, the respondent’s subjective financial situation, the respondent’s employment situation and if the respondent lives in a big city, in the suburbs or outskirts of a big city, in a town or a small city, in a country village, farm or in the countryside.

#### Country-Level Variables

As an indicator of income inequality we use the country level Gini index because this is the measure that is typically used in the literature to characterize the relationship between income inequality and generalized trust (see for example Alesina and La Ferrara, 2002; Uslaner and Brown, 2005; Gustavsson and Jordahl, 2008). The Gini index is a summary measure representing how income is distributed in a country. The Gini index ranges between 0 and 100, where 0 represents perfect equality – everyone enjoys the same income – and 100 represents perfect inequality – a single individual controls all the economic resources available to a community. We use data on the Gini index for the year in which the survey was implemented. Data on the Gini coefficient come from the World Bank, Development Research Group (For more information and methodology, please see PovcalNet: http://iresearch.worldbank.org/PovcalNet/index.htm).

As an indicator for the economic situation in the country and living standards in which respondents live, we use an indicator of per capita GDP for the year in which the survey was administered (OECD, 2019a). Because the cost of living is different in different countries and different countries use different currencies we use a standardized measure of per capita GDP (which reflects purchasing power parity) and is expressed in US dollars.

Finally, migration flows (as measured by the change in the number of foreign-born individuals who reside in a country between the study year t and t-2) and the percentage of people born in a foreign country as an indication for the overall level of migration. Data on migrant flows come from OECD migration statistics (OECD, 2018) while percentage of people born in the foreign country was derived using ESS data themselves.

In the multilevel analysis we standardize all of the country level variables to have a mean zero and standard deviation of one performing *z*-score standardization.

### Modeling

#### Measurement Invariance and Scaling the Main Constructs

Our data involves individuals surveyed in eighteen European countries in one of the four last rounds of the European Social survey (2010, 2012, 2014, and 2016) on a number of indicators that can be used to describe individuals’ opposition to migration and feelings of threat. Therefore, while our data allow us to examine differences across countries and survey years, this is possible only if measurement invariance (also referred to as measurement equivalence in the literature) is established. Meaningful comparisons of means or associations like covariances and unstandardized regression coefficients across countries and time points can only be conducted in the presence of measurement equivalence (Meredith, 1993; Steenkamp and Baumgartner, 1998; Davidov et al., 2014; Pokropek et al., 2017).

The classical approach to test measurement invariance is Confirmatory Factor Analysis (CFA) modeling and Multiple Group Confirmatory Factor Analysis (MG-CFA). CFA and MG-CFA assume that the observed indicator *Y*_*ig*_ is continuous and the relation between the latent trait η_*j*_ for an individual j and observed indicators *Y*_*ig*_ is described by a linear relation (for a simple one-dimensional case):

(1) $$y_{i\,g}=\tau_{i\,g}+\lambda_{i\,g}\,\eta_{j\,g}+\varepsilon_{i\,g}$$

where *τ*_*ig*_ describes the factor intercept while *λ*_*ig*_ indicates the factor loading of the item *i* in group *g*. Where *e*_*ig*_ denotes a random error.

A scale is said to be configurationally invariant when the measurement CFA model of different groups has the same structure. Configurational invariance determines whether respondents in different groups use the same conceptual framework when they answer particular survey questions (Horn and McArdle, 1992; Vandenberg and Lance, 2000; Cheung and Rensvold, 2002). In practice, configural invariance is assumed to be achieved if the fit of CFA model for each of analyzed group is sufficiently high (usually measured by CFI > 0.9 and REMSA < 0.05 indices). Passing a configurational invariance test is a necessary, but not a sufficient condition to draw valid comparisons of relations between the analyzed scale and other variables. Such comparisons can be conducted only in the presence of metric equivalence (or weak factorial invariance), i.e., when the loadings of indicators on the factors are equal across respondents in different groups. By ensuring that metric equivalence is respected we can say that respondents in different groups interpret the intervals on the same response scales and that the estimated latent constructs tap into the same underlying concept. Testing for metric equivalence consists of testing the model fit of a MG-CFA model where factor loadings for the indicator *i* are restricted to be the same across all groups. If the MG-CFA with such restrictions holds and fits the data, metric equivalence is considered to be achieved (Vandenberg and Lance, 2000; Cheung and Rensvold, 2002).

The presence of configural and metric invariance does not allow to conduct meaningful comparison of means of the underlying constructs. In order to do so, full comparability has to be established. Scalar invariance (or strong factorial invariance) established full comparability and requires that both factor loadings and the intercepts for each item used in the construction of a scale should be the same across groups (in our case countries and survey years). Scalar invariance is established by examining the change in the model fit indices. In the presence of scalar invariance, respondents can be said to use the same scale origin and have the same anchor in all groups. Scalar invariance allows for valid comparisons of means of latent variables across groups (Cheung and Rensvold, 2002; Schaffer and Riordan, 2003).

Researchers often experience difficulties in achieving full metric and scalar invariance, especially when the number of groups is large or when cultural differences are significant. This was also the case in our study: full scalar and metric invariance was not achieved. However, comparisons across groups can be reliably made in contexts when only a subset of indicators functions equivalently. This situation is referred to as partial equivalence (Byrne et al., 1989; Steenkamp and Baumgartner, 1998). A recent simulation study show that even with one invariant item per scale, under certain conditions, valid comparisons can be performed (Pokropek et al., 2019). We applied the concept of partial measurement invariance to our data. We used the sequential method used in alignment optimization proposed by Asparouhov and Muthén (2014) to detect non-invariance of parameters. We follow the conclusions of simulation studies which indicate that alignment procedures provide the optimal combination of Type I error control and power (Finch, 2016). All parameters flagged as non-invariant were estimated freely in the subsequent analysis. We assume that at least one non-invariant factor loafing per group could correctly identify metric invariance and additionally one non-invariant factor intercept could identify scalar invariance.

On top of partial invariance, we checked the level of approximate measurement invariance (AMI), to establish if small differences in factor loadings and item intercepts across different groups exist. AMI postulates that small differences between groups are a ubiquitous and inevitable consequence of between-group comparisons. As such they would not prevent comparisons but, rather, such small deviations should be incorporated into the statistical modeling using Bayesian methods, using the so-called Multi-Group Bayesian Structural Equation Modeling (MG-BSEM, Muthén and Asparouhov, 2013). In MG-BSEM models, “elastic” equality constraints are introduced to relax the assumption of full invariance (Braeken and Blömeke, 2016; Seddig and Leitgoeb, 2018; Lek et al., 2019). We tested different models using different level of elasticity, starting with exact priors and increasing the elasticity of constraints. We use a stepwise model selection strategy, starting from a model with a zero difference prior (exact constraint), and compared this model with a higher difference prior (i.e., 0.001), i.e., introducing more elastic constraints. We then compared the fit of the model with the higher prior (i.e., 0.001) to the one with the exact constraint. If model fit was better (DIC > 3 for cross-time comparison and DIC > 10 for cross-country comparisons), we continued the exercise and compared the higher prior model with one with an even higher prior (i.e., 0.005). We started the procedure from a 0.000 prior (i.e., exact invariance) and evaluated seven different priors that define different levels of elasticity for the item parameters constraints: 0.001, 0.005, 0.010, 0.015, 0.020, 0.025 and 0.050. Elastic constraints were applied only to the parameters that were not flagged as partially non-invariant.

We performed analyses of measurement invariance as follows: for cross-time invariance we tested each country separately and for cross-country invariance, we tested each survey wave separately. The aim of this two-step procedure was identify the primary source of non-invariance, country differences of differences between survey waves. We used the results obtained for those countries that exhibited at least cross-country and cross-time metric invariance to estimate a combined model and generate factor scores. Final factor scores were generated using Bayesian estimation of MG-CFA models with exact constraints (since the analyses we conducted indicated that the level of small differences was negligible).

#### Modeling the Relations Between Education and Opposition to Migration

**FIGURE 1:**
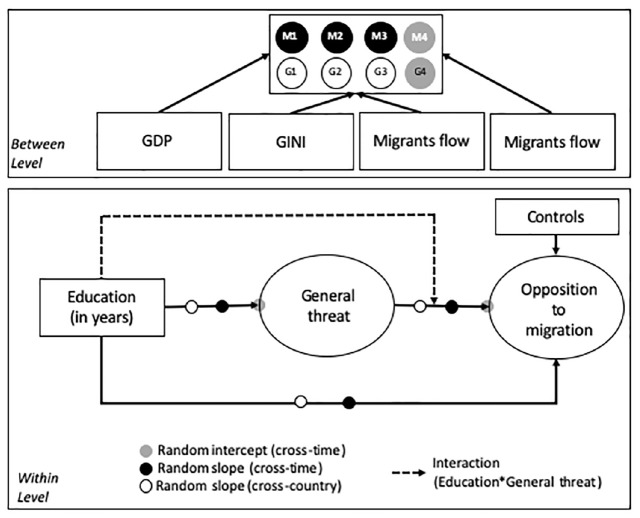
Theoretical mediation model of the association between education and opposition to migration.

Figure 1 illustrates the hypothesized pathways between education and opposition to migration, including both the direct relationship between education and opposition to migration as well the indirect relationship mediated by feelings of general threat, a latent construct.

Our analyses were aimed to achieve three objectives. First, we were interested in identifying the relationship between education and opposition to migration and the role of a general threat as a mediation and moderation variable. In order to establish this baseline relationship we assumed homogeneity of relationships across countries and time points, effectively estimating our model on the pooled sample of individuals from different countries and survey waves and constraining model parameters to be the same irrespective of the country of residence and survey period. Second, we relaxed the assumption of homogeneity of model parameters and explore if the relations between variables differed substantially between countries and between years (variation parameters are depicted by black and white dots) and whether the level of measured constructs differed across time points (gray dots) inside each country. Because our measurement invariance analysis indicated that that we lack sufficient information to compare the means of the variables across countries, we could only examine variations in means between time points within each country. We did so by standardizing the different latent scales to have a mean of 0 and a SD of 1 within each country in year 2010. The standardized values of index X for country *c* can be defined as follows: Z_c_ = (X_c_-M_cX__2010_)/SD_cX__2010_ where M_cX__2010_ is the mean of the index in year 2010 for respondents form country *c* and year 2010 and SD_X__2010_ is the standard deviation of the index for country c in year 2010. Finally, we examined if differences in relationships were systematic and were determined by the following four country-level variables: GDP per capita, the Gini index, the % of migrants and the change in such percentage in the 2 years prior to the survey administration.

To achieve our goals we employed three modeling strategies. First, we estimated a simple mediation moderation model on a pooled dataset for all countries and all data points to have an overview of the average results. Then we developed a cross-classified multilevel model (Luo, 2017) where the variance of the random components was decomposed into cross-country and cross-time components (depicted as dots in Figure 1). The cross-classified model provided us with information on how much of the variance in the random parameters in the pooled dataset should be attributed to differences between countries and how much to differences across survey years. Due to the complexity of the model, we tested each random component and its two potential sources of variation one at a time. For instance, in the second model we examined the variation of the relation between education and general threat and decomposed this variation into cross-time and cross-country parts, while in the following model we tested the variation of the relation between general threat and opposition to migration and its two potential sources in cross-time and cross-country differences. Finally, in the last modeling step, we used multilevel mediation moderation modeling (Preacher et al., 2007; Hayes, 2013) where the group was defined both by year in which the survey was conducted and the country in which it was conducted to explain the overall variation in random parameters using time and country varying variables. Initially we tested a set of different mediation -moderation multilevel models, checking for whether conflated effects across levels of analysis occur (Preacher et al., 2016). However, because we did not find any evidence for such effects, we opted for a simple multilevel approach based on multilevel path analysis (Bauer et al., 2006). All models was estimated in Mplus 8 using Bayes estimation with non-informative priors. This choice was dictated by the complexity of the cross-classified models and the better performance of Bayes estimation for mediation effects (in particular the fact that this procedure provides correct estimates of standard errors (Wang and Preacher, 2015).

## Results

### Comparability of Attitudes Toward Migration and General Threat

#### Comparability Over Time

Table 1 illustrates results of analyses that we carried out to assess the comparability over time of the opposition to migration index, while Table 2 illustrates results of analyses on the comparability of the general threat index. For each index, we denote individual items using the integers 1, 2, and 3. In Table 1 “people of the same race or ethnic group as most [country] people to come and live here?” is referred to with the number 1, “how about people of a different race or ethnic group from most [country]?” is referred to with the number 2 and “people from the poorer countries in Europe?” is refereed to with the number 3. Similarly, for Table 2, economic threat is referred to with the number 1, cultural threat is referred to with the number 2 and prejudice is refereed to with the number 3. Plain numbers indicate that a particular indicator in a country and year was comparable with the same indicator in the same country in the other survey years both in factor loadings and slopes. A parenthesis () indicates lack of invariance of factor loadings and brackets [] indicate lack of invariance of factor slopes. Last two columns indicate the level of approximate non-invariance (i.e., the small overall differences between items that were not flagged as non-invariant).

**TABLE 1:** Invariance analysis of the opposition to migration index.

**Country**	**Year**												**Approx. MI**	
		
	**2010**			**2012**			**2014**			**2016**			**Loadings**	**Intercepts**
Belgium	1	2	3	1	2	3	1	2	[3]	1	2	3	0.001	0.001
Switzerland	1	2	3	1	2	3	1	2	[3]	1	2	3	0.001	0.001
Czechia	1	2	3	[1]	2	[3]	1	2	2	1	2	3	0.000	0.000
Germany	[(1)]	2	3	1	[2]	3	1	2	[3]	1	[2]	3	0.000	0.000
Estonia	1	2	3	1	2	3	1	[2]	3	1	2	[3]	0.001	0.001
Spain	1	2	3	1	2	[3]	[1]	2	[3]	1	[2]	3	0.000	0.000
Finland	1	[2]	3	1	2	(3)	1	[2]	[3]	1	2	3	0.000	0.001
France	[(1)]	2	(3)	1	2	3	1	2	3	1	[2]	[3]	0.000	0.000
Great Britain	1	2	3	1	2	3	1	[2]	[3]	1	2	3	0.001	0.001
Hungary	[1]	[2]	3	[1]	[2]	3	1	2	3	1	2	[(3)]	0.000	0.000
Ireland	1	2	3	1	2	3	[1]	2	[3]	1	[2]	3	0.000	0.000
Israel	1	2	3	1	2	3	[1]	2	[3]	[1]	[2]	3	0.005	0.001
Lithuania	1	2	3	[1]	(2)	3	1	2	[3]	1	[(2)]	3	0.001	0.000
Netherlands	1	2	3	1	2	3	1	[2]	[3]	1	2	3	0.000	0.001
Norway	1	2	3	1	2	3	1	2	3	1	2	[3]	0.001	0.001
Poland	1	2	3	1	2	3	1	2	[3]	1	[2]	3	0.000	0.000
Portugal	[1]	2	[3]	1	2	3	1	2	3	1	[2]	3	0.001	0.001
Slovenia	1	2	3	1	2	3	[1]	2	[3]	[1]	[2]	3	0.001	0.000

*1,2,3 item number; [] indicates non-invariance in intercepts; () indicates non-invariance in factor loadings.*

**TABLE 2:** Invariance analysis of the general threat index.

**Country**	**Wave**												**Approx. MI**	
		
	**2010**			**2012**			**2014**			**2016**			**Loadings**	**Intercepts**
Belgium	1	2	3	1	2	3	1	2	3	1	2	3	0.001	0.001
Switzerland	1	2	3	1	2	3	1	2	3	1	2	3	0.000	0.000
Czechia	[1]	2	3	1	2	3	1	2	3	[1]	2	3	0.000	0.001
Germany	1	2	[3]	1	2	3	1	2	3	[1]	[2]	3	0.001	0.001
Estonia	1	2	3	1	2	3	1	2	3	1	(2)	3	0.001	0.001
Spain	1	2	3	1	2	3	1	2	3	1	2	3	0.001	0.000
Finland	1	2	3	1	2	3	1	2	3	1	2	3	0.001	0.001
France	1	2	3	1	2	3	[1]	2	3	1	2	3	0.001	0.001
Great Britain	1	2	3	[1]	[2]	3	1	2	3	[1]	2	3	0.000	0.001
Hungary	1	2	3	1	2	3	1	2	3	1	[(2)]	3	0.001	0.001
Ireland	[(1)]	[2]	3	[1]	2	3	1	2	3	1	2	3	0.000	0.001
Israel	1	2	[3]	1	2	(3)	1	2	3	1	2	3	0.000	0.001
Lithuania	[1]	2	3	1	2	3	1	2	3	1	2	3	0.001	0.001
Netherlands	1	2	3	1	2	3	[1]	2	3	1	[2]	3	0.001	0.000
Norway	1	2	3	1	2	3	1	2	3	1	2	[3]	0.000	0.001
Poland	1	2	3	1	2	3	1	2	3	1	[2]	3	0.001	0.001
Portugal	1	2	3	1	2	3	1	2	3	1	2	3	0.005	0.005
Slovenia	1	2	3	1	2	3	1	2	3	1	2	3	0.001	0.001

*1,2,3 item number; [] indicates non-invariance in intercepts; () indicates non-invariance in slopes.*

Results presented in Table 1 suggest that some items are partially non-invariant (showing lack of invariance in either intercepts or factor loading) and a small number of items are approximately non-invariant (showing lack of invariance in both intercepts and factor loadings). Simulation studies indicate that scales with one non-invariant item per group can yield reasonably reliable measures, particularly when the level of non-invariance is small (in our case 0.001 which is negligible given standard benchmarks of 0.005) (Pokropek et al., 2019). Table 2 illustrates similar results for general threat. Overall findings presented in Tables 1, 2 suggest that it is possible to construct scales of opposition to migration and general threat that are fully comparable within countries over time.

#### Between Country Comparability

Tables 3, 4 illustrate results of invariance testing for the extent to which the opposition to migration and general threat items are comparable across countries within a single wave of data. As for Tables 1, 2, we denote individual items using the integers 1, 2, and 3 and use the same number for the same items. Plain numbers indicate that a particular indicator was fully comparable both in factor loadings and slopes. A parenthesis () indicates lack of invariance of factor loadings and brackets [] indicate lack of invariance of factor slopes. Last two rows indicate the level of approximate non-invariance.

**TABLE 3:** Opposition to migration index, between country invariance.

**Country**	**Year**											
	
	**2010**			**2012**			**2014**			**2016**		
Belgium	[1]	[2]	3	[1]	[2]	3	1	2	3	[1]	[2]	3
Switzerland	**(1)**	**[(2)]**	**(3)**	[1]	[2]	3	**[(1)]**	**(2)**	**(3)**	[(1)]	2	(3)
Czechia	[1]	[(2)]	3	(1)	[(2)]	[3]	1	(2)	[3]	[1]	2	3
Germany	1	[2]	3	[1]	[2]	[3]	[(1)]	2	3	1	2	(3)
Estonia	[(1)]	2	[(3)]	[1]	[2]	[3]	[(1)]	[2]	[3]	(1)	[2]	[3]
Spain	[1]	[(2)]	[3]	**[(1)]**	**[(2)]**	**[(3)]**	[1]	[(2)]	[3]	[(1)]	[(2)]	[3]
Finland	[1]	2	[3]	[1]	[2]	[3]	[(1)]	[2]	[3]	[(1)]	[2]	3
France	[1]	(2)	3	[1]	[(2)]	3	1	[(2)]	3	[1]	2	3
Great Britain	[1]	(2)	3	1	(2)	(3)	1	[(2)]	3	[1]	[2]	3
Hungary	[1]	2	[3]	[1]	[2]	[3]	[1]	[2]	[3]	1	2	[(3)]
Ireland	[1]	(2)	3	(1)	[(2)]	3	1	[(2)]	3	[(1)]	(2)	[3]
Israel	[(1)]	[2]	[3]	[1]	[2]	[(3)]	[(1)]	[2]	[(3)]	[(1)]	(2)	3
Lithuania	1	2	[3]	[(1)]	[(2)]	[3]	[1]	[2]	[(3])	[1]	[2]	[3]
Netherlands	[(1)]	[(2)]	3	1	(2)	(3)	**[(1)]**	**[(2)]**	**(3)**	[1]	[2]	3
Norway	[(1)]	[2]	[3]	[1]	[2]	3	(1)	2	3	[1]	2	[3]
Poland	[1]	[(2)]	[3]	(1)	[(2)]	[3]	1	[(2)]	[3]	(1)	[(2)]	[3]
Portugal	[(1)]	(2)	[3]	(1)	(2)	3	[1]	[(2)]	[3]	[1]	2	[3]
Slovenia	[1]	(2)	3	(1)	(2)	3	1	[(2)]	3	[1]	2	3
Approx. MI (load)	0.001			0.001			0.001			0.001		
Approx. MI (Int)	0.001			0.001			0.000			0.001		

*1,2,3 item number; [] indicates non-invariance in intercepts; () indicates non-invariance in factor loadings, in bold occasions where all factor loadings were flagged as non-invariant and those occasions were excluded from the modeling phase.*

**TABLE 4:** General threat index, between country invariance.

**Country**	**Year**											
	
	**2010**			**2012**			**2014**			**2016**		
Belgium	1	[2]	3	[1]	2	3	1	2	3	[1]	2	[3]
Switzerland	[1]	2	3	[1]	2	[3]	1	[(2)]	[(3)]	[1]	[2]	[3]
Czechia	[1]	2	3	[1]	[2]	3	1	(2)	3	[1]	[2]	3
Germany	[(1)]	[2]	[3]	[1]	2	[(3)]	[(1)]	2	3	[(1)]	2	[(3)]
Estonia	[1]	[2]	[3]	[1]	2	[3]	[(1)]	[2]	[3]	[1]	2	[3]
Spain	1	[2]	3	1	2	[3]	(1)	[(2)]	[3]	1	[(2)]	[3]
Finland	1	[(2)]	3	1	[(2)]	[(3)]	[(1)]	[2]	[3]	1	[(2)]	3
France	[1]	[2]	3	[1]	[(2)]	[3]	1	[(2)]	3	1	2	3
Great Britain	[1]	2	3	[1]	[2]	3	(1)	[(2)]	3	[1]	[2]	[(3)]
Hungary	1	[2]	3	(1)	[(2)]	[3]	[1]	[2]	[3]	1	2	3
Ireland	[(1)]	[2]	[(3)]	1	[2]	[(3)]	1	[2]	3	1	[(2)]	[(3)]
Israel	1	2	[3]	[1]	[2]	(3)	[(1)]	[2]	[(3)]	1	2	3
Lithuania	1	2	3	[1]	[2]	3	[1]	[2]	[(3)]	[1]	[2]	3
Netherlands	1	[2]	3	1	2	3	**[(1)]**	**[(2)]**	**(3)**	1	2	3
Norway	[1]	2	3	[1]	[2]	[3]	1	2	[3]	1	[2]	3
Poland	1	[2]	[3]	1	2	3	1	[(2)]	[3]	1	[2]	[(3)]
Portugal	[(1)]	[2]	[3]	[(1)]	2	[3]	[1]	[(2)]	[3]	1	2	[3]
Slovenia	1	[2]	3	1	2	3	1	[(2)]	3	[1]	2	3
Approx. MI (load)	0.001			0.001			0.010			0.010		
Approx. MI (Int)	0.001			0.001			0.005			0.010		

*1,2,3 item number; [] indicates non-invariance in intercepts; () indicates non-invariance in factor loadings in bold occasions where all factor loadings were flagged as non-invariant and those occasions were excluded from modeling phases.*

Contrary to findings presented in Tables 1, 2, results presented in Tables 3, 4 indicate that it is not possible to construct scales that are fully comparable across countries. In many countries all intercepts for each of the three indicators of opposition to migration and general threat are non-invariant. However, factor loadings are mostly invariant. Only in 4 country-year combinations all factor loadings are estimated to be non-invariant (Switzerland in 2010, Switzerland in 2014, Spain in 2012, and the Netherlands in 2014).

Because most factor loadings are invariant, results presented in Tables 3, 4 can be interpreted as follows: it is possible to construct scales for opposition to migration and general threat to be used to investigate differences in relations between latent variables across countries as a function of certain characteristics, but cannot be used to establish reliable mean rankings across countries. Tables 3, 4 also suggest that the level of approximate non-invariance is higher when we attempt to establish comparability across countries within a survey wave than when we attempt to establish comparability within a country across survey waves (as we do in Tables 1, 2). Formal tests of cross-country comparability range between 0.001 and 0.005 (with noticeably higher value for loadings in 2014). Although these values are higher than those estimated in the context of invariance testing for the comparability of indicators within a country over time, they are small enough to allow the construction of latent variables provided that proper statistical techniques are used.

### Comparing Changes Over Time Within Countries

Using information from the comparability analysis, we scaled the latent variables “opposition to migration index” and “general threat index.” The two scales are fully comparable across years in each county but only metric invariance was established between countries in each survey wave. This means that although it is possible to compare trends in the mean index of opposition to migration and general threat in one country across time, we cannot compare the mean index values in a single wave across countries. However, because we were able to establish metric invariance, we can compare the relationship between the two indices and other variables both across countries and across time.

**FIGURE 2:**
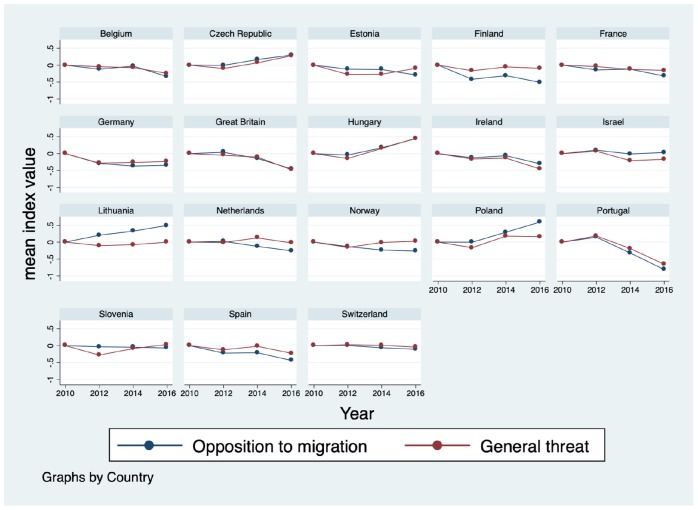
Country specific changes in opposition to migration and general threat.

Figure 2 illustrates, for each country with available data, the evolution over time in the opposition to migration index and the general threat index. Because mean levels of the indicators cannot be compared across countries, we show country specific results. Detailed results reporting significance testing for changes across survey waves are available in Supplementary Appendix Tables A1, A2. In the majority of countries, changes in the extent to which individuals oppose migration go hand in hand with changes in levels of perceived threat. In Belgium, the Czech Republic, Germany, Great Britain, Hungary, Ireland, Portugal, Spain, and Switzerland the pattern of changes in the two indices is remarkably similar. By contrast, in Lithuania, Norway, Estonia, Poland, and Slovenia changes in opposition to migration differ from changes in individuals’ perceptions of general threat. In particular, in many of these countries patterns appear to diverge between 2014 and 2016.

#### The Association Between Education and Attitudes Toward Migration: The Role of General Threat

Table 5 presents results of the baseline model that we estimated using the pooled sample of countries and survey years. The baseline model allows us to identify the extent to which the association between education and opposition to migration is direct or is mediated by individuals’ feelings of threat, under the strict constraint of homogeneity of relations across countries and over time. Crucially, because we were able to establish metric invariance of our latent constructs, we are able to interpret and compare direct and indirect associations across countries and over time. Table 5 indicates that around 60% of the overall association between education and opposition to migration is indirect: individuals who attended school for longer tend to be less opposed to migration than individuals who attended school for fewer years because they report lower feelings of threat and such feelings are importantly associated to how opposed to migration an individual is. Table 5 also report the extent to which education moderates the association between feelings of threat and opposition to migration. Although this is statistically significant at conventional level, it is quantitatively very small.

**TABLE 5:** Baseline model examining the association between education and opposition to migration.

**Parameter**	**Estimate**	**Posterior *SD***	**One-Tailed *P*-Value**	**Lower 2.5% CI**	**Upper 2.5% CI**
Opposition←Education	−0.090^∗^	0.003	<0.001	−0.096	−0.084
Opposition ←Threat	0.471^∗^	0.002	<0.001	0.466	0.476
Threat ← Education	−0.308^∗^	0.004	<0.001	−0.315	−0.301
Moderation effect of Education	0.008^∗^	0.002	<0.001	0.004	0.013
Indirect effect of Education	−0.145^∗^	0.002	<0.001	−0.149	−0.141
Total effect of education	−0.235^∗^	0.005	<0.001	−0.242	−0.229

*Control variables in Appendix. DIC = 548167.630. ^∗^Significant at 0.05 level.*

In Table 6 we relax the assumption of homogeneity of associations across countries and time points. We fit cross-classified models to examine the extent to which differences in the association between education and opposition to migration varies across individuals depends on the country and the year in which they were surveyed. Although the number of groups and timepoints observed in our study is limited, similar research and simulation studies show that reliable results can be obtained in these conditions (see Schmidt-Catran and Fairbrother, 2015). In Model 1 (M1) we allow the intercept of the opposition to migration index and the general threat index to vary across time points. Because our invariance analyses indicated that our measures reached metric but not full invariance, we scaled the two measures to be comparable overtime within a country but not across countries, i.e., in each country the two variables were scaled to have a mean of zero and a standard deviation in year 2010. The variance across time appears to be significant with random effects of 0.091 for opposition to migration and 0.095 for threat. Both random intercepts were statistically significant. By comparing the Deviance Information Criterion (DIC) measure in M1 (when we introduce random intercepts to allow opposition to migration and threat to vary over time) and the estimates in the baseline model, the model fit improves considerably (544414.705 compared with 548167.630 in the baseline model). These results highlight the significant variation within countries over time of the two variables. These results match the graphical representation displayed in Figure 2, which highlighted an important variation over time in both opposition to migration and feelings of threat.

**TABLE 6:** Decomposition of random effects between countries and time points.

**Estimates**	**M1 random intercepts (opposition, threat)**	**M2 opposition ↑ education**	**M3 threat ↑ education**	**M4^†^ opposition ↑ threat**	**M5 moderation (random)**
Main effect	—	−0.101^∗^	−0.324^∗^	0.491^∗^	0.004
SD (Country)	—	0.032^∗^	0.117^∗^	0.088^∗^	0.032^∗^
SD (Time)	0.091^∗^	0.019^∗^	0.044^∗^	0.023^∗^	0.059^∗^
	0.095^∗^				
DIC	544414.705	544343.558	543682.437	544792.105	544239.373

*^∗^Significant at 0.05 level; ^†^Convergence problems. Models with control variables.*

In Models 2, 3, and 4 we estimate random slopes in our model, effectively relaxing the assumption of homogeneity in associations across countries and time points. We establish whether relaxing the homogeneity of associations assumption is warranted by examining the size of the random effect, the level of significance level and the increase in model fit, as measured using DIC. We proceed by modeling one random slopes at a time as follows: in model M2 we examined the variation in the direct association between education and opposition to migration. In M3 we examined the variation in the association between education and feelings of threat and in M4 we investigated the variation in the association between feelings of threat and opposition to migration. Finally, in model 5 we examined the variation in the moderation effect of education on the relationship between feelings of threat and opposition to migration.

Overall, results presented in Table 6 indicate that the underlying associations depicted in Figure 1 between education and opposition to migration differ both across countries and over time. In particular, results suggest that the random slopes estimated in models 2, 3, and 4 which consider variations in the direct association between education and opposition to migration and the association between education and feelings of threat and between feelings of threat and opposition to migration are quantitatively meaningful, statistically significant and yield better fitting models. Moreover results of model 5 indicate that although the average effect of moderation is close to zero this effect varies across countries, such that that in the most extreme settings in our sample it is positive while in others it is negative and is, therefore, difficult to interpret correctly.

#### The Influence of Social Context in Explaining Differences Across Countries and Over Time in the Association Between Education and Attitudes Toward Migration: Mediation Moderation Analyses

Although results presented in Table 6 support the notion of differences in underlying associations between education, feelings of threat and opposition to migration both over time and across countries, they cannot be used to identify if such differences are systematic and are related to the context experienced by individuals. In Table 7 we examine if the level of income inequality (as indicated by the Gini coefficient), living standards (as indicated by per capita GDP), the level of diversity present in a country (as indicated by the percentage of residents who are foreign born), and the change in diversity (as indicated by the change in the percentage of foreign-born residents), explain between country differences and differences over time in underlying relationships.

**TABLE 7:** Cross-classified mediation-moderation model: factors that explain the variation across countries and survey years in estimated relationships.

**Slopes explained by**		**Estimate**	**Posterior *SD***	**One-Tailed *P*-value**	**Lower 2.5% CI**	**Upper 2.5% CI**
Opposition ← Education	GDP	0.015^∗^	0.009	0.030	−0.001	0.032
	FLOWS	−0.006	0.006	0.162	−0.017	0.005
	GINI	0.012	0.009	0.102	−0.005	0.030
	% of MIG	−0.014	0.010	0.084	−0.033	0.005
Threat ← Education	GDP	0.007	0.020	0.366	−0.034	0.045
	FLOWS	−0.008	0.014	0.318	−0.038	0.020
	GINI	0.037	0.022	0.056	−0.011	0.080
	% of MIG	−0.057^∗^	0.023	<0.001	−0.105	−0.016
Opposition ← Threat	GDP	0.052^∗^	0.016	0.002	0.023	0.084
	FLOWS	−0.010	0.012	0.178	−0.035	0.012
	GINI	0.013	0.017	0.204	−0.018	0.049
	% of MIG	−0.044^∗^	0.018	0.006	−0.082	−0.012
Moderation	GDP	−0.004	0.007	0.298	−0.017	0.010
	FLOWS	0.003	0.005	0.282	−0.006	0.013
	GINI	−0.006	0.008	0.214	−0.022	0.008
	% of MIG	0.000	0.008	0.486	−0.015	0.016

*^∗^Significant at 0.05 level.*

Results presented in Table 7 indicate that the level of birthplace diversity present in a country in a specific year is significantly associated with the education gradient in feelings of threat: in particular, in the presence of more foreign-born residents the education gradient appears to be steeper which translates directly into stronger indirect and total effects (see Supplementary Appendix Figure A1). However, in the presence of greater birthplace diversity feelings of threat appear to be less strongly associated with how opposed to migration individuals are. Overall these results suggest that, although a higher percentage of foreign-born individuals results in more polarized attitudes toward migration with better educated individuals expressing considerably lower feelings of threat than those who attended schooling for a smaller number of years, such attitudes do not necessarily translate in greater opposition to migration because feelings of threat are less associated with support for restrictive migration legislation in countries with more foreign-born residents. By contrast, in countries and survey years in which living standards are higher, feelings of threat are more strongly associated with how opposed individuals are to migrants.

## Discussion

Education is often considered an important element to foster openness to diversity and ensure that individuals do not perceive migration phenomena as a threat (Schneider, 2008). However, much less is known about the mechanisms that facilitate education’s role in promoting favorable attitudes toward migration and, in particular, how individuals with different levels of education react to changes in their economic and social environment. Behavioral theories of decision-making such as the theory of reasoned action (Fishbein and Ajzen, 1975) and the theory of planned behavior (Ajzen, 1988, 1991; Ajzen and Fishbein, 2005) emphasize how intentions are the proximate antecedents of behavior and, in turn, how attitudes, norms and perceived behavioral control are the proximate antecedents of behavioral intentions. In fact, there is (limited) empirical evidence based on longitudinal data for Germany and Russia on the causal effect of feelings of threat on intended behavior toward migrants (Schlueter et al., 2008).

Examining if and how education influences the attitudes individuals express toward migration phenomena and how this influence is shaped by the social contexts in which individuals operate is important to understand, and potentially influence, their intended behavior and, with it, their behavior. Attitudes can therefore shape the intention individuals have to support and vote for anti-immigration and nationalistic political parties or, by contrast, to vote for political parties that see migration, if coupled with effective integration policies, as an economic and cultural opportunity. They may also shape the intention individuals have engage with local NGOs and civil societies organizations to ensure that integration policies are available for foreign-born individuals or, by contrast, to engage in protests designed to pressure politicians to adopt restrictive migration legislation.

We examined data from the last four waves of the European Social Survey to identify the changing association between education and attitudes toward migration in European countries between 2010 and 2016, a period of rapid changes in the economic and social landscape in Europe. In particular, in 2015 a very large number of refugees and asylum seekers fleeing conflict entered Europe at a time when many European societies were just emerging from the protracted economic crisis that followed the collapse of financial institutions in 2008.

We concentrate on two factors that characterize individuals’ preferences for restrictive migration policies and their feelings of economic threat, cultural threat and prejudice that have been extensively used in the literature. Invariance testing reveals that the two latent constructs, opposition to migration and feelings of general threat, can be compared within countries over time. However, because we are able to establish metric invariance but not scalar invariance, we are able to compare across countries associations between factors but not the mean levels of the two indicators. These results should be considered in conjunction with other work on the cross-country comparability of preferences for restrictive migration policies and feelings of economic threat, cultural threat and prejudice in Europe (see for example Coenders et al., 2005).

Results reveal a large degree of heterogeneity in the evolution of opposition to migration and feelings of threat in different European countries between 2010 and 2016. In the majority of countries preferences for restrictive migration policies and feelings of threat evolve in similar ways, although in Lithuania, Norway, and Poland trends in the evolution of the two factors differed in the most recent years.

Our analyses indicate that education was strongly associated with how opposed to migration individuals in Europe reported to be, with better educated individuals expressing lower levels of opposition than poorly educated individuals. As much as 60% of differentials in opposition to migration between individuals with different levels of education can be ascribed to the indirect channel of better educated individuals feeling lower levels of general threat, and in turn, the strong association between levels of threat and opposition to migration. Our results reveal a high degree of heterogeneity in underlying associations both across countries and over time. We attempted to identify if such heterogeneity can be explained by living standards, levels of income inequality, the presence of foreign-born populations and how such presence evolved in the two years prior to the interview. We find that the presence of foreign-born populations explains differences across countries and over time in the association between education and feelings of threat: in the presence of greater numbers of migrants, educational differentials in feelings of threat are greater. However, we also find that feelings of threat are less strongly associated with opposition to migration in the presence of more foreign-born residents in a country. By contrast, feelings of threat appear to be more associated with opposition to migration when living standards are higher.

Our results are consistent with theories that consider education as an important determinant of attitudes toward migration because of the influence it has on feelings of economic threat, cultural threat and prejudice that individuals experience in response to the presence of foreign-born individuals (Schneider, 2008; Borgonovi, 2012; d’Hombres and Nunziata, 2016). However, given the very specific geographical context and timeframe under investigation, both methodological (small number of time points per unit) and substantive considerations (specificity of conditions) mean that findings should be investigated further and validated.

The finding that there is a marked difference in the extent to which highly educated and poorly educated individuals report being opposed to migration phenomena suggests that although what happens in classrooms can play a positive role in strengthening social cohesion in the presence of foreign-born populations by equipping individuals with skills and cultural awareness, disparities in educational opportunities and attainment can create highly polarized public opinions on topics of increasing social and political relevance. The fact that individuals with greater educational attainment experience lower threat suggests that even if individuals can be open to the social and cultural diversity that results from migration flows, at the moment formal education is the primary channel that helps develop the cognitive capacity, emotional dispositions and psychological states that are necessary to not feel threatened by the presence of foreign-born populations. Furthermore, as technological innovation changes both the types of skills that are demanded and rewarded in the workplace, a number of authors have argued that as the speed and intensity of technological progress is increasing, transformation may have disruptive consequences for workers (Brynjolfsson and McAfee, 2011; Mokyr et al., 2015). Fears of widespread technological unemployment may be overstated, but the impact of digital transformations on the nature of work and skills requirements may exacerbate feelings of economic threat, particularly among individuals with low levels of qualifications and skills (OECD, 2019b). The political and social significance of this finding cannot be underestimated since, unless remedied, a profound cultural gap between social classes is likely to emerge.

Moving forward, it is important that education systems and schooling will equip all individuals, not only those who obtain higher level qualifications, with the ability to either not feel threatened by the culture of new arrivals or with the ability to respond positively to feelings of threat such that they do not lead individuals to hold discriminatory views and attitudes. Potential actions include on fostering global competencies early on in the school years to ensure that all individuals, irrespective of their eventual highest educational attainment, will develop similar levels of the foundation skills that are necessary to be open and understand different cultures and traditions. Education systems in many countries are increasingly aiming to foster global competence in their students, enabling them to appreciate different perspectives and world views, and interact successfully and respectfully with others (OECD, 2018). In order to ensure that older cohorts are not left behind, the development of lifelong learning programs could be developed in order to help older cohorts with the knowledge and skills that are necessary to be able to understand multicultural issues and deal with the tensions they create in everyday life.

## Data Availability Statement

Publicly available datasets were analyzed in this study. This data can be found here: https://www.europeansocialsurvey. org/data.

## Author Contributions

FB designed the study, identified the relevant theoretical framework, and drafted the manuscript. AP designed the study, analyzed the data, and drafted the manuscript.

## Conflict of Interest

The authors declare that the research was conducted in the absence of any commercial or financial relationships that could be construed as a potential conflict of interest.
