# Supplementary material for: Education and Attitudes Toward Migration in a Cross Country Perspective
Source: Front Psychol. 2019 Oct 18;10:2224. doi: 10.3389/fpsyg.2019.02224 (PMC6842942; doi:10.3389/fpsyg.2019.02224)
Supplement: Supplementary file 1 [file Table_1.pdf]

Table A1.

Mean results of attitudes toward migration (comparable across time, not comparable across countries)

	2010	2012		2014		2016	
Country	mean	mean	se	mean	se	Mean	se
Belgium	0.00	**-0.122**	(0.041)	-0.029	(0.044)	**-0.337**	(0.043)
Switzerland	0.00	0.005	(0.049)	-0.062	(0.049)	**-0.106**	(0.051)
Czech Republic	0.00	-0.008	(0.040)	**0.164**	(0.036)	**0.298**	(0.036)
Germany	0.00	**-0.290**	(0.038)	**-0.368**	(0.034)	**-0.344**	(0.039)
Estonia	0.00	**-0.120**	(0.041)	**-0.130**	(0.043)	**-0.289**	(0.050)
Spain	0.00	**-0.221**	(0.042)	**-0.209**	(0.041)	**-0.437**	(0.041)
Finland	0.00	**-0.419**	(0.046)	**-0.311**	(0.046)	**-0.508**	(0.046)
France	0.00	**-0.142**	(0.046)	**-0.120**	(0.050)	**-0.323**	(0.051)
Great Britain	0.00	0.041	(0.041)	**-0.143**	(0.046)	**-0.449**	(0.042)
Hungary	0.00	-0.043	(0.051)	**0.171**	(0.049)	**0.444**	(0.049)
Ireland	0.00	**-0.123**	(0.036)	-0.062	(0.036)	**-0.301**	(0.037)
Israel	0.00	0.103	(0.060)	-0.010	(0.056)	0.035	(0.047)
Lithuania	0.00	**0.205**	(0.054)	**0.326**	(0.054)	**0.494**	(0.061)
Netherlands	0.00	0.022	(0.044)	**-0.124**	(0.042)	**-0.257**	(0.043)
Norway	0.00	-0.131	(0.044)	**-0.233**	(0.046)	**-0.266**	(0.045)
Poland	0.00	-0.003	(0.040)	**0.295**	(0.044)	**0.593**	(0.046)
Portugal	0.00	0.145	(0.044)	**-0.323**	(0.050)	**-0.811**	(0.051)
Slovenia	0.00	-0.033	(0.050)	-0.046	(0.049)	-0.069	(0.049)

Note: bolded numbers indicate resultssignificantly difrent from 0 (year 2010) at $p<0.05$
