# Supplementary material for: Education and Attitudes Toward Migration in a Cross Country Perspective
Source: Front Psychol. 2019 Oct 18;10:2224. doi: 10.3389/fpsyg.2019.02224 (PMC6842942; doi:10.3389/fpsyg.2019.02224)
Supplement: Supplementary file 2 [file Table_2.pdf]

Table A2.

Mean results of general threat (comparable across time, not comparable across countries)

	2010	2012		2014		2016	
Country	mean	mean	se	mean	se	mean	se
Belgium	0.00	-0.047	(0.046)	-0.075	(0.047)	**-0.241**	(0.046)
Switzerland	0.00	0.027	(0.056)	0.012	(0.055)	-0.043	(0.056)
Czech Republic	0.00	**-0.101**	(0.042)	0.068	(0.039)	**0.276**	(0.040)
Germany	0.00	**-0.281**	(0.037)	**-0.264**	(0.037)	**-0.232**	(0.040)
Estonia	0.00	**-0.274**	(0.046)	**-0.269**	(0.048)	**-0.097**	(0.048)
Spain	0.00	**-0.121**	(0.046)	-0.019	(0.043)	**-0.224**	(0.044)
Finland	0.00	**-0.167**	(0.037)	-0.053	(0.040)	**-0.093**	(0.041)
France	0.00	-0.043	(0.046)	**-0.127**	(0.049)	**-0.154**	(0.045)
Great Britain	0.00	-0.047	(0.045)	**-0.103**	(0.045)	**-0.473**	(0.046)
Hungary	0.00	**-0.144**	(0.043)	**0.151**	(0.044)	**0.454**	(0.048)
Ireland	0.00	**-0.159**	(0.041)	**-0.134**	(0.039)	**-0.452**	(0.039)
Israel	0.00	0.071	(0.061)	**-0.207**	(0.052)	**-0.169**	(0.057)
Lithuania	0.00	-0.103	(0.061)	-0.075	(0.058)	-0.003	(0.060)
Netherlands	0.00	-0.011	(0.048)	**0.125**	(0.052)	-0.021	(0.052)
Norway	0.00	**-0.156**	(0.048)	-0.011	(0.051)	0.028	(0.053)
Poland	0.00	**-0.165**	(0.044)	**0.178**	(0.048)	0.161	(0.048)
Portugal	0.00	**0.185**	(0.056)	**-0.191**	(0.070)	-0.656	(0.069)
Slovenia	0.00	**-0.280**	(0.050)	-0.085	(0.050)	0.034	(0.052)

Note: bolded numbers indicate resultssignificantly difrent from 0 (year 2010) at $p<0.05$
