# Supplementary material for: Education and Attitudes Toward Migration in a Cross Country Perspective
Source: Front Psychol. 2019 Oct 18;10:2224. doi: 10.3389/fpsyg.2019.02224 (PMC6842942; doi:10.3389/fpsyg.2019.02224)

Figure A1.

Total and indirect effects of education on opposition to migration and % of foregin-born population.

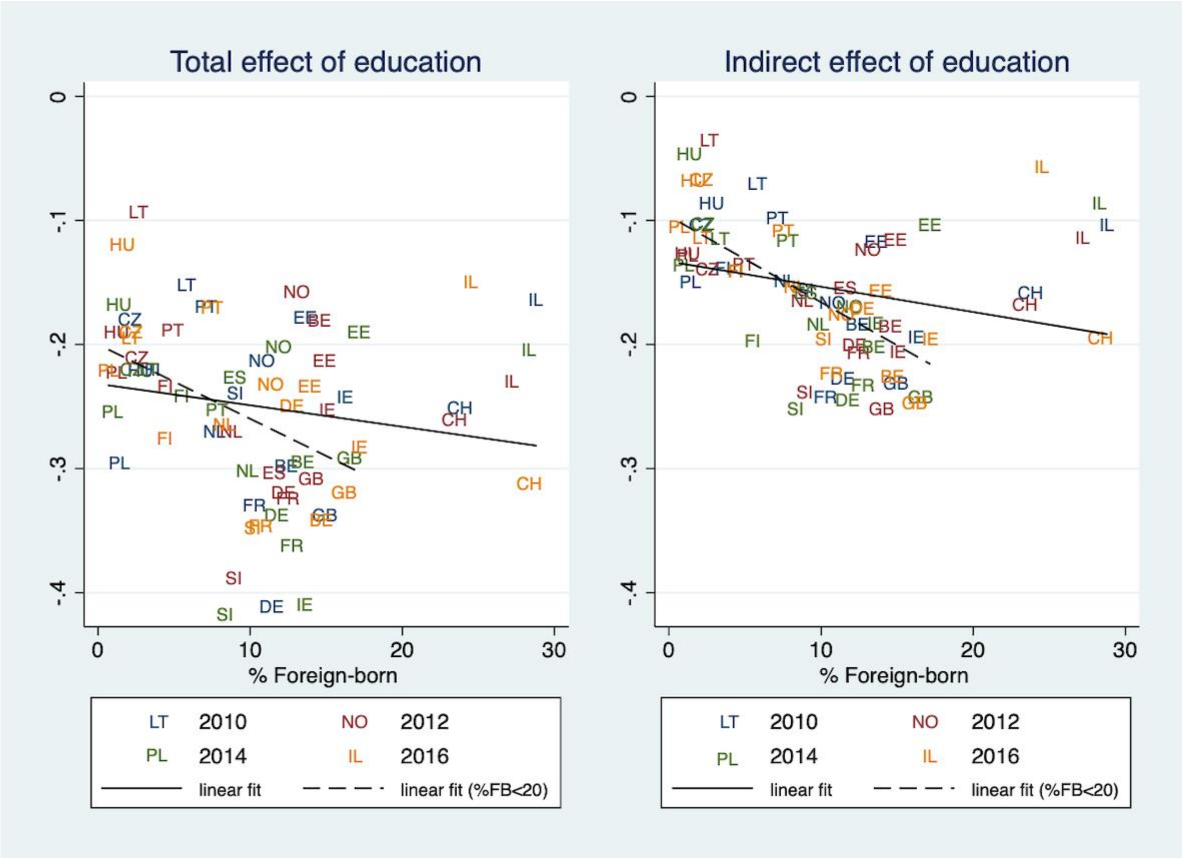

Supplement: Supplementary file 3 [file Image_1.pdf]
